# Experimental study of ASCs combined with POC-PLA patch for the reconstruction of full-thickness chest wall defects

**DOI:** 10.1371/journal.pone.0182971

**Published:** 2017-08-11

**Authors:** Yuanzheng Zhang, Shuo Fang, Jiezhi Dai, Lei Zhu, Hao Fan, Weiya Tang, Yongjie Fan, Haiying Dai, Peipei Zhang, Ying Wang, Xin Xing, Chao Yang

**Affiliations:** 1 Department of Plastic Surgery, Changhai Hospital, Second Military Medical University, Shanghai, PR China; 2 Department of Orthopedic Surgery, Shanghai Jiao Tong University Affiliated Sixth People's Hospital, Shanghai, PR China; 3 State Key Laboratory for Modification of Chemical Fibers and Polymer Materials, College of Materials Science and Engineering, Donghua University, Shanghai, PR China; 4 Department of Plastic Surgery, the 455th hospital of Chinese People's Liberation Army, Shanghai, PR China; Università degli Studi della Campania "Luigi Vanvitelli", ITALY

## Abstract

To explore the repairing effect of combination of adipose stem cells (ASCs) and composite scaffolds on CWR, the electrospun Poly 1, 8-octanediol-co-citric acid (POC)-poly-L-lactide acid (PLA) composite scaffolds were prepared, followed by *in vitro* and *in vivo* biocompatibility evaluation of the scaffolds. Afterwards, ASCs were seeded on POC-PLA to construct the POC-PLA-ASCs scaffolds, and the POC-PLA, POC-PLA-ASCs, and traditional materials expanded polytetrafluoroethylene (ePTFE) were adopt for CWR in New Zealand white (NZW) rabbit models. As results, the POC-PLA-ASCs patches possessed good biocompatibility as the high proliferation ability of cells surrounding the patches. Rabbits in POC-PLA-ASCs groups showed better pulmonary function, less pleural adhesion, higher degradation rate and more neovascularization when compared with that in other two groups. The results of western blot indicated that POC-PLA-ASCs patches accelerated the expression of VEGF and Collagen I in rabbit models. From the above, our present study demonstrated that POC-PLA material was applied for CWR successfully, and ASCs seeded on the sheets could improve the pleural adhesions and promote the reparation of chest wall defects.

## Introduction

Intact thoracic wall plays an important role in breathing movement and protects the indispensable content. The full-thickness defects of the chest well commonly caused by tumor resection, trauma, infection, burn, and radiation [1,2]. Flaps have been developed for soft tissue reconstruction depend on the location on the chest wall, size of the defects, arc of rotation of the flap, and availability of recipient vessels. Bony reconstruction is performed with mesh or mesh combining with rib substitutes according to the number of defect ribs [3].

Optimal material for chest wall reconstruction (CWR) remains controversial. Typical mesh in clinical CWR was permanent synthetic mesh, including polypropylene and expanded polytetrafluroethylene [4,5]. While complications were associated with these material s during the post-operation follow-up, such as infection, fracture, contraction and insufficient tensile strength. Furthermore, surgical removal is inevitable when mesh infection was occurred. Bioabsorbable mesh materials, such as poly-L-lactide acid (PLA) and polydioxanone are another choice with better resistance to infection and greater implant-defect interface strength [6,7]. For rigid materials, it could afford maintenance of chest wall stability, but the lack of resilience affects the movement of the chest wall during breath, moreover the pleural adhesion between lung and mess [8]. Significant adhesions occurred in patient with thoracic surgeries account for the recurrent thoracotomies and high risk of bleeding, and the prolonged air leaks [9,10].

Poly 1, 8-octanediol-co-citric acid (POC) is reported as a biodegradable elastomer with controllable mechanical and mild inflammatory response, which has potential for use in tissue engineering [11,12]. Synthesis of POC can be conducted under very mild condition without addition of toxic catalysts, which stimulating inspiration of combination of POC with drugs or protein, such as growth factor-delivering microparticles [13]. POC-PLA composite scaffolds were widely applied in various tissue reconstruction. Electrospun composite scaffolds containing Poly (octanediol-co-citrate) are 3-dimensional elastomeric substrates potentially beneficial for CWR [14,15], while the effects of electrospun POC-PLA composite scaffolds on CWR need more explorations.

Sheet-based bioengineering concept is a non-invasive harvest of cultured cells as intact tissue sheets using polymer-coated culture surfaces [16]. Adipose tissue represents an important source of adult stem cells, called ASCs (adipose stem cells) [17], which is abundant in the body. Adipose stem cells (ASCs) can be harvested as sheet-like structures with significant amount of deposited extracellular matrix (ECM) after long-term culture in temperature-responsive culture surfaces. ASCs are tremendous interest to regenerative medicine [17, 18]. ASCs cell sheet instead of standard cell culture techniques would remain the ECM and cytokines, which can seed the cells at high density, and reduce pleural adhesions and accelerate the CWR, and further reinforce the full-thickness excisional wounds regeneration through paracrine effects [19, 20].

Thus, we hypothesized that the electrospun POC-PLA composite scaffolds seeded with ASCs cell sheet may reduce the pleural adhesion and improved the pulmonary function after CWR in New Zealand white (NZW) rabbit models.

## Materials and methods

### Ethics statement

All animal experiments were approved by the Changhai Hospital affiliated to Second Military Medical University and conducted according to the Animal Care Committee of Shanghai Changhai Hospital. All surgery was performed under sodium pentobarbital anesthesia, and every effort was made to minimize suffering.

### Scaffold fabrication

The electrospun synthesis scaffold was carried out according to literature reports [15]. Equimolar amounts of citric acid (ACS reagent, 99.5%, Sigma-Aldrich, Steinheim, Germany) and 1,8-Octanediol (98%, Aldrich, Steinheim, Germany) were taken in a three-neck round bottom flask, and then were under magnetic stirring in an inert atmosphere of nitrogen at 160°C for 10 min. The temperature slowly lowered to 140°C and kept for 40 min to increase the degree of cross-linking. Then the mix liquor was transferred to the beaker and washed several times by ethanol and distilled water to harvest purer POC.

Equal definite proportions of POC and PLA (Yisheng materials Co., Ltd, Shenzhen, China. 10% mass fraction) were dissolved in trifluoroethanol, and then was thorough stirred by magnetic stirrer to ensure it was completely dissolved. The liquid was then transferred into a 5 ml syringe, after few minutes' standing, it was put on the pushing device with the connection of the needle and the positive pole of electrostatic generator. Afterwards, set the flow velocity as the solution was flowed smoothly from the needle, then powered on the electrostatic generator and adjust to the appropriate voltage to spinning. When the spinning is finished, subsequently put the aluminum film covered with film sheets in a vacuum drying oven for solidification.

### Scaffold characterization, mechanical testing and degradation

The morphology and diameter of the fibrous scaffolds were determined via the scanning electron microscope (SEM). Before observed, the samples were fixed, dried and metallized. The images were selected randomly for calculation of average diameter and porosity. The porosity was measured as the ratio of the void space within the material to its total displacement volume, and it was evaluated via Image J software (NIH, Bethesda, USA) in this study. Patches were cut into size of 3*1 cm, and fixed by retention clips of mechanical sensors for mechanical testing.

The *in vitro* degradation rates were detected via PBS method. Firstly, patches were cut into size of 3×3 cm and the initial weight were recorded after dry for 3 days. The patches were then soaked in 20 ml PBS and placed in the cell incubator, afterwards, the samples were weighed monthly from 1 month to 7 months later as degradation weight. Then draw a weight curve to calculate the *in vitro* degradation rates.

### Biocompatibility detection

Mixed cultivation of the I-929 rat fibroblasts and the patches were conducted to evaluated the biocompatibility of the patches *in vitro*, in which assay, the cell proliferation was detected by the method of MTT, the cells cultured in same medium were used for control. Briefly, the POC-PLA patches were sterilized and were then put in the 24-well plates for 20 min at 37°C with 200 μl RPMI 1640 medium. Afterwards, the I-929 rat fibroblasts were trypsinized, seeded and cultured in 24-well plates with the density of 3000–5000 cells per plate. Then culture solution was added and the patches with cells were cultured in the incubator. For MTT, cells were washed with PBS for twice, and re-washed by the serum-free medium. Afterwards, 360 μl serum-free medium and 40 μl MTT solution were added and mixed. Then the plates were covered by aluminum foil and incubated for 1 h in incubator at 37°C. After incubation, supernatants were removed, and 400 μl of DMSO were added in each plate and mixed for 30 min. Afterwards, 100 μl liquids from each plate was transferred to 96-well plates and the absorbance was recorded at 595 nm. The percent viability was expressed as absorbance in the presence of test compound as a percentage of that in the vehicle control.

The biocompatibility of the patches *in vivo* was detected via subcutaneous implantation experiment in rats. 21 healthy SD rats (male, weighted 170–200 g) were randomly divided into 3 groups: group A (Treated with patches for 1 w), group B (Treated with patches for 4 w), and group C (4 weeks after sham-operated group as control). Briefly, the adult SD rats were anesthetized by intraperitoneal injection of sodium pentobarbital (30 mg/kg, from Shanghai solarbio Bioscience & Technology Co., LTD) and were fixed on the plate in a supine position next, then sheared and disinfected via iodophor and 75% alcohol. The abdominal midline skin of rats were sliced, the skin and peripheral connective tissue were separated by scissor to form the subcutaneous pockets. The sterilized POC-PLA electrostatic spinning patches were cut into squares with size of 1×1 cm, and then were stuffed into the subcutaneous pockets after soaked in PBS for 2 h. The rats in control groups were treated without patches. Afterwards, the incision was screwed up and the rats were maintained under specific pathogen free (SPF) conditions with antibiotics taken daily. 1 and 4 weeks later, the rats were sacrificed and the samples were collected for future immunological histological chemistry (IHC) experiment. For IHC, briefly, sections were deparaffinized and rehydrated. Antigen unmasking was done by steaming in EDTA buffer (pH 0.8) for 10 min. Endogenous peroxidase activity was blocked by incubating in 3% hydrogen peroxide solution for 10 min. Then, primary antibodies were allowed to react in dilutions of 50 μl overnight at 4°C. After washing in PBS, a horseradish peroxidase-labeled polymer (50–100 μl) was then applied for 50 minutes at 4°C. Peroxidase was visualized by 3, 30-diaminobenzidine tetrahydrochloride (DAB) as the chromogen. After rinsing in de-ionized water and counterstaining in Harris’ hematoxylin, the slides were dehydrated and mounted. The antibodies used for IHC were anti-CD68 antibody (Abcam, ab955), anti-IL6 antibody (Abcam, ab9324), anti-TNF-α antibody (Abcam, ab220210), anti-TGF beta antibody (Abcam, ab190503), anti-Ki67 antibody (Abcam, ab15580), anti-PCNA antibody (Abcam, ab18197), anti-Caspase-3 antibody (Abcam, ab13847), and in situ Apoptosis Detection Kit (Abcam, ab206386).

### ASCs isolation and culture

Rabbit subcutaneous adipose tissue was acquired from the inguinal fat pads of female New Zealand White (NZW) rabbits (4–5 month, 2–2.5 kg) in sterile surgical room. After washed 3–4 times with PBS to remove the blood clots on the surface, tissue samples were dissected away the hypodermal layer and phanerous vessels. Then transferred to petri dish, and carefully minced into tiny pieces around 1 mm^3^. 0.1% collagenase type II (Sigma-Aldrich, St. Louis, USA) was added to digest the fat under mild agitation for 45 min at 37°C. After centrifuged at 800 g for 10 min, the floating tissue was removed and the pellets were resuspended and pass through 70-μm nylon mesh filters. The filtrate was then centrifuged at 500 g and washed twice with PBS. The final cell pellet were resuspended in Dulbecco’s modified Eagle’s medium (DMEM) supplemented with 10% fetal bovine serum, 100mg/ml streptomycin, and 100 U/ml penicillin (from Amresco, Solon, OH, USA) and cultured in a 5% carbon dioxide (CO_2_) humidified incubator at 37°C. After 24 h the growth medium was removed with floating cells and new medium was added. Then the medium was replaced 3–4 days depending on the cell growth and the remained cell population was represented as passage 1. Then the cell-surface phenotype were identified by flow cytometry as described before [21]. The antibodies used for flow cytometry were as follows: anti-Integrin beta 1 (CD29) antibody (Abcam, ab78502), anti-CD31 antibody (Abcam, ab199012), anti-CD34 antibody (Abcam, ab213058), anti-CD44 antibody (Bio-Rad, MCA806GA), anti-CD45 antibody (Bio-Rad, MCA808GA), anti-CD73 antibody (eBioscience, PA5-47628), anti-CD90 (Abcam, ab225), anti-CD105 antibody (Abcam, ab11415).

### Sheet preparation and characterization

1×10^6^ passage 3 ASCs were seeded onto a temperature-responsive cell surface plate (Thermo Scientific Nunc Upcell Surface) to create the cell sheet. After cultured for 24 h until 100% fusion rate, all medium was aspirated and a fibrin-coated membrane was gently placed over the cell sheet in the thermoresponsive dishes at 20°C for 10 min. Then membrane was transferred to the electrospun scaffold with the attached cell layer facing downwards. The construct was moved to the incubator for 30 min at 37°C. Then the membrane was gently withdrawn with the cell layer attached on the electrospun mesh. Afterwards, the membrane with cell layer were cultured in cell incubator for 24 h. SEM micrographs were taken to observe the cell morphology and the density on the scaffold.

### Surgical procedure

Total of 45 NZW rabbits (2–2.5 kg) were randomly divided into 3 groups: the expanded polytetrafluoroethylene (ePTFE) mesh group, the POC-PLA electrospun mesh attaching with ASCs sheet as ASCs group and POC-PLA electrospun mesh group. All animals were fed a diet of standard rabbit chow and water ad libitum.

The rabbit was anesthetized with sodium pentobarbital (30 mg/kg intravenously) and maintained with ketamine hydrochloride (2 mg/kg intravenously). After intratracheal intubation, the rabbit was placed in the operating room table in the left lateral decubitus position. The right lateral chest wall was shaved and sterilized with povidone iodine solution. A 5-cm incision was made parallel to the seventh rib. The latissimus dorsi and the serratus anterior muscles were split and separated from the ribs. A 3×3 cm full-thickness chest wall defect was created by resecting the sixth through eighth ribs including the costal muscle and underlying parietal pleura. After completion of the hemostasis, the 3.5×3.5 cm POC-PLA electrospun mesh attaching with ASCs sheet was interposed into the defect and sutured into place. The latissimus dorsi muscle, subcutaneous tissue and skin were stratified closed. In other two groups, expanded polytetrafluoroethylene (ePTFE) mesh and POC-PLA electrospun mesh were respectively used to repair the defect. The endotracheal tube was removed after the rabbit resumed breathing spontaneously. 40000 U penicillin a day was subcutaneously injected in the following 3 days.

The weight and operation complications were followed up until 24 weeks or until sacrifice.

### Pulmonary function detection

The pulmonary function of rabbits were detected via AniREs 2005 Animal respiratory function test system on pre-operation and the 4^th^, 12^th^ and 24^th^ week postoperatively. The detection index were as follows: forced expiratory volume of 0.4 and 0.6 secs (FEV_0.4_ and FEV_0.6_), forced vital capacity (FVC), and 25–75% forced expiratory flow (FEF25-75%), and then FEV_0.6_/FVC% and FEV_0.4_/FVC% were calculated.

### Graft harvest and pleural adhesions

The rabbits were sacrificed at 4 week, 12 week, 24 week postoperatively (n = 5 rabbits each per time point per group). The repair sites were examined for thoracocyllosis and lung herniation. Then the grafts were resected with 2 cm peripheral normal tissue, and the adhesion between graft and intrapleural structures was graded from 0 to 5 in increments of 0.5 as reported [22]. In this system, normal pleura was scored 0; pleura with tough surface or obvious inflammatory was scored 1; pleura with adhesive band easy to separate and less than 3 was scored 2; score 3, adhesive band > 3 or hard to separate; score 4, obvious adhesive band > 5; pleura with thick adhesive bands which can’t be separated was scored 5. Then the sample was sectioned as 3×1 cm to test the tensile strength and the right normal thoracic wall as a control.

### Histologic and immunohistochemical analysis

All samples were equally sectioned into two pieces, one for formalin-fixed paraffin, and other for cryo-section. The paraffin specimens were sectioned 5 μm and the cryo-section 6–8 μm for histologic evaluations. H&E, masson’s trichrome, and Elastic Van Gieson (EVG) were used to evaluate the tissue formation under manufacturer's instruction. Moreover, immunohistochemical staining for CD31 was performed. The antibody was anti-CD31 antibody (Abcam, ab199012).

### Western blot

Rabbit tissues were lysed using a protein lysis buffer containing 20 mM Tris (pH 7.4), 150 mM NaCl, 1 mM EDTA, 1 mM EGTA, 1% Triton X-100, 25 mM sodium pyrophosphate, and 2 mM sodium orthovanadate aprotinin. Total proteins were separated by SDS-PAGE gel and transferred to polyvinylidene difluoride membranes (Roche, USA). The membranes were blocked with 5% skimmed milk in Tris-buffered saline and then incubated with primary antibodies as follows: anti-VEGF, anti- Collagen I and anti-Tubulin. The samples were then incubated with horseradish peroxidase-conjugated anti-rabbit secondary antibody. The bands were visualized using the ECL Western Blotting Kit (Biovision, USA) and quantified by Image J software. The polyclonal antibodies VEGF, Collagen I, and β-tublin (used at a dilution of 1:50) used for Western blot in this study were all purchased from R&D Systems China Co. Ltd, China.

### Statistical analysis

Data were presented as mean ± standard deviation (SD). ANOVA was used to determine statistical significance among experimental groups. A p-value < 0.05 was considered statistical significance.

## Results

### Generation and characterization of the electrospun POC-PLA composite scaffolds

**Fig 1:**
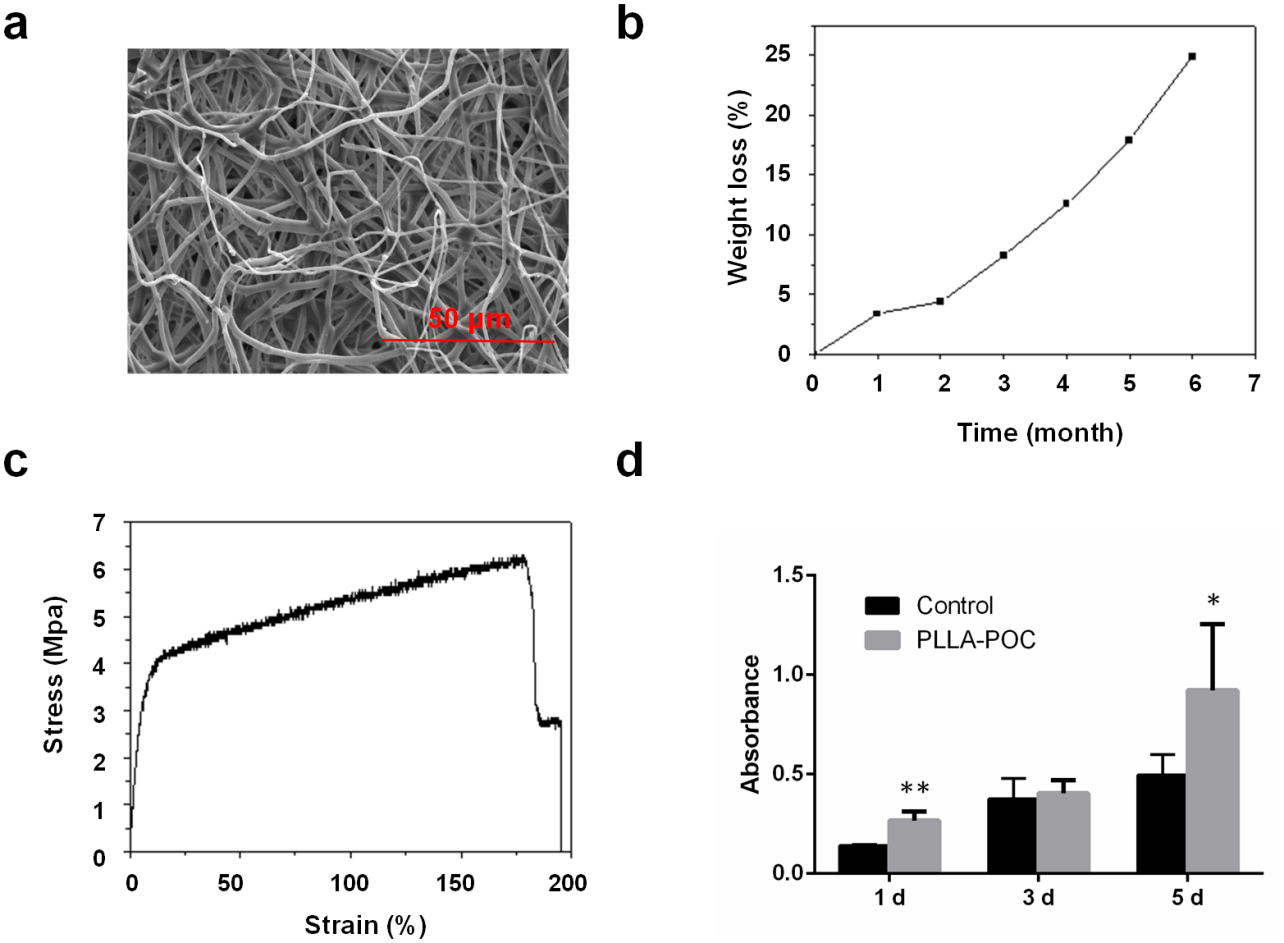
characterization of the electrospun POC-PLA composite scaffolds. (a) Representative SEM images of POC-PLA composite scaffolds. (b), and (c) showed the *in vitro* degradation rates and result of mechanical testing of POC-PLA scaffolds. (d) The cell proliferation of I-929 rat fibroblasts co-cultured with the patches was detected by the method of MTT (cells cultured in same medium were used for control). *, P<0.05; **, P<0.01.

The POC-PLA electrospun fibrous patches were white membranes about 2 mm thick and possessed the characteristics of smooth and opaque. As shown in Fig 1a, SEM evaluation of POC-PLA electrospun fibrous patches showed that the POC-PLA composed by thin strip fibers with numerous pores. 20 fibers were randomly selected to calculate the average diameter, which was 1.429 μm. As results, the porosity was 86.4%. 20 pores were randomly selected, and the mean diameter was 18.01 μm.

The average rate of in vitro degradation of POC-PLA electrospun fibrous patches was 24.86% semiannually. The tensile strength of POC-PLA electrospun fibrous patches was 6.3 MPa and its elongation at break point was 179.9% (Fig 1b and 1c).

### Biocompatibility evaluation of the electrospun POC-PLA scaffolds

The results of MTT at 1, 3 and 5 day post operation indicated that the numbers of fibroblasts were similar with cell numbers in control group, while the cell numbers on POC-PLA patches were more than that in control group (As shown in Fig 1d), especial at the 1^st^ and 5^th^ day post operation, the differences were significant. The rat’s diet, activity were observed until 4 weeks after surgery, no obvious changes were found.

**Fig 2:**
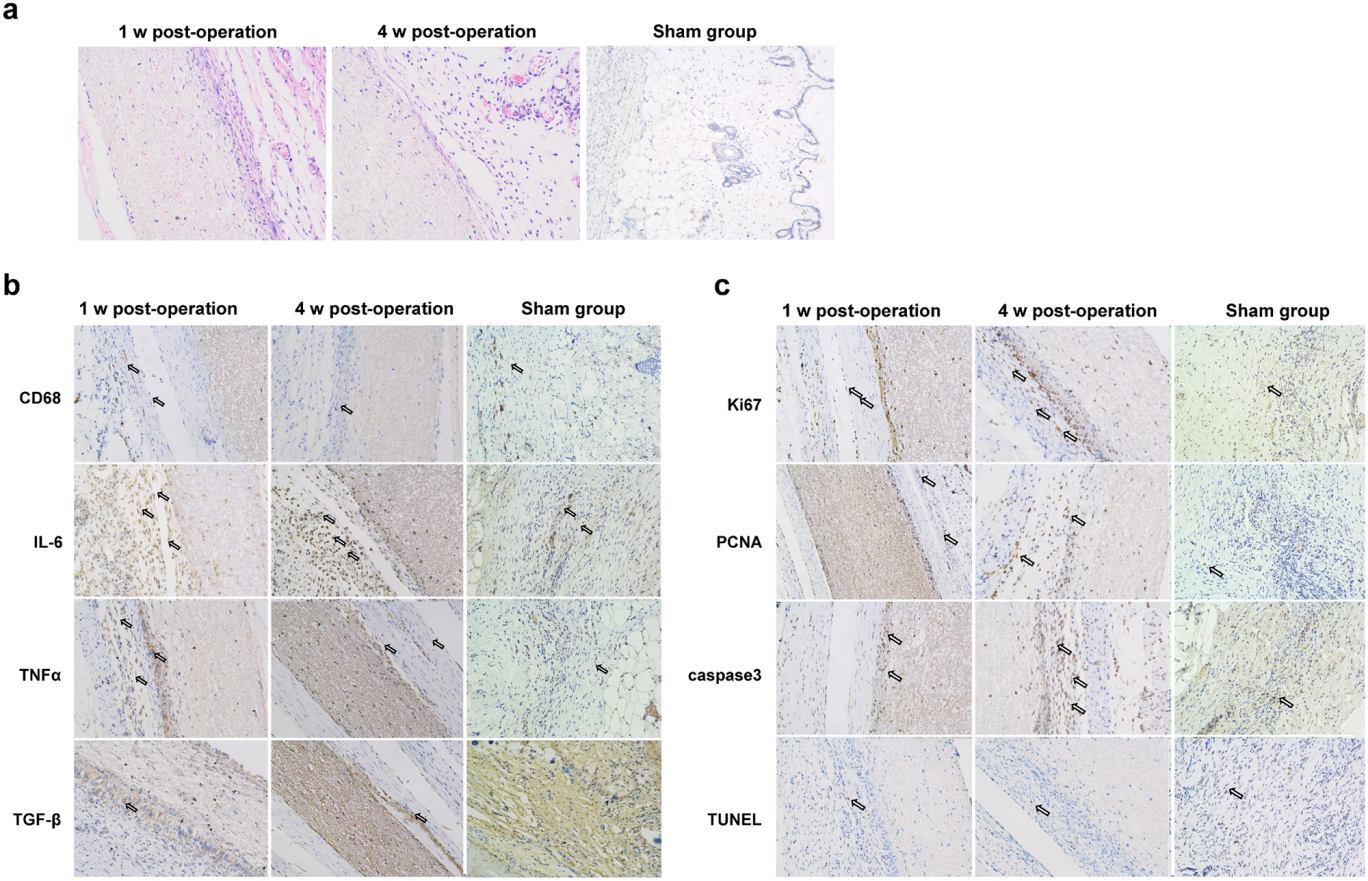
*In vivo* biocompatibility Detection of POC-PLA patches. (a) The representative H&E staining results in rats from different groups (magnification 100×). (b), and (c) shows the representative images of IHC in each group (magnification 100×). Sham, 4 weeks after sham-operated group as control.

At the first week, PLA-POC was loosely covered with tissues, and the surrounding tissue showed mild inflammation and blood vessel is filled well. The PLA-POC patches connected with surrounding tissues firmly and no obvious inflammation and tissue proliferation was observed at the 4^th^ week after surgery. Meanwhile, H&E staining results in Fig 2a showed the red blood cells were observed in the patches.

The results of IHC showed that few CD68 positive cells were observed around the implants at 1 week and 4 weeks after surgery. TNF-α, IL-6, and TGF-β were commonly expressed in cells, which were highly expressed in experimental group when compared to control group 1 week after surgery, while were similar to control group at the 4^th^ week (Fig 2b).

IHC staining of Ki67 and PCNA in Fig 2c also indicated the strong proliferation ability of cells around implants. IHC results also showed darker and denser staining of capsase-3 in cells around patches at 1^st^ and 4^th^ weeks after surgery. Tunel staining revealed few apoptotic cells around implants at 1^st^ week after surgery, and was fewer at 4 weeks post-operation, which was stay low levels in sham operation group.

### ASCs isolation and sheet preparation

**Fig 3:**
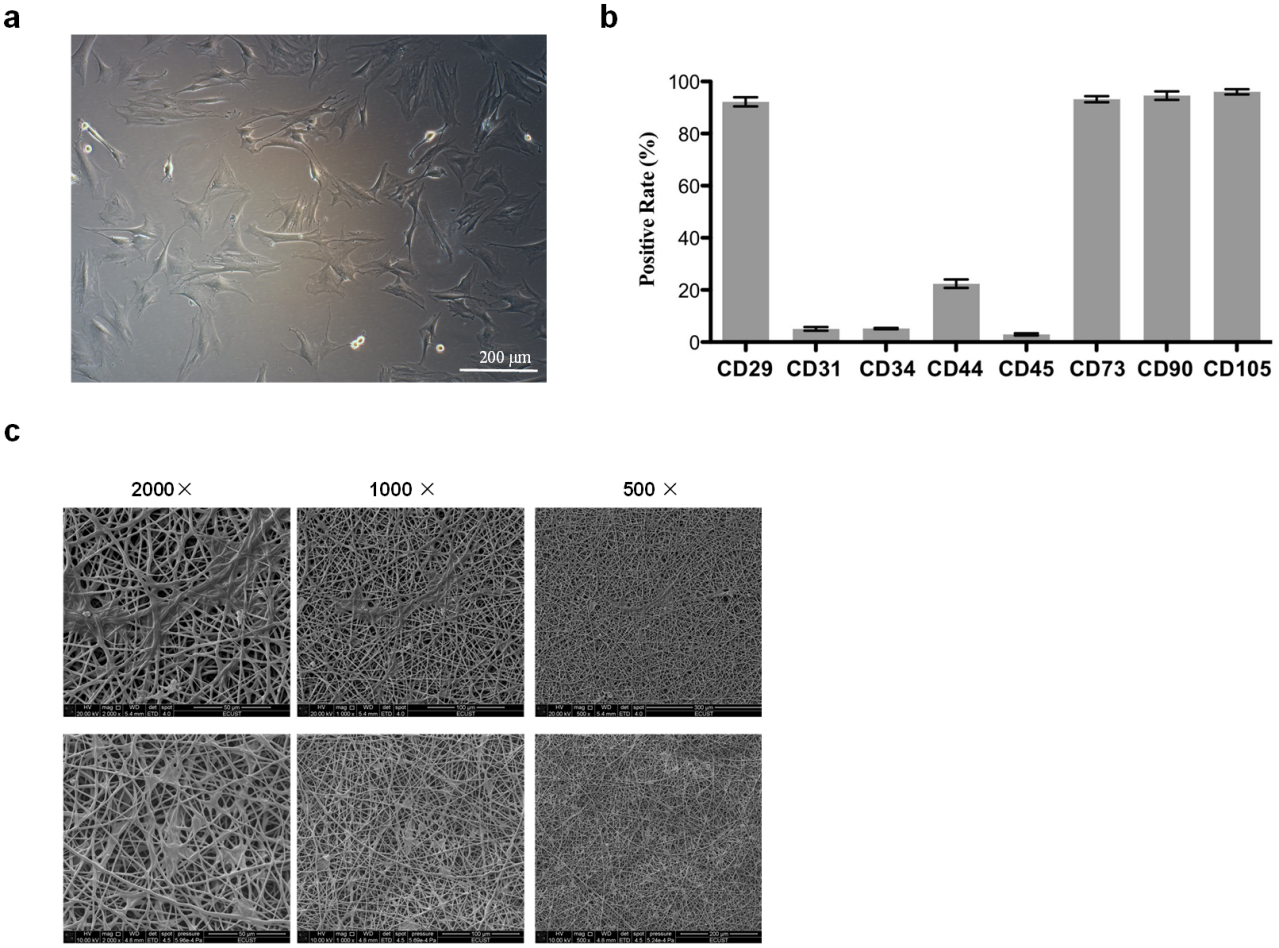
Isolation and identification of ASCs. (a) The second generation ASCs (with fusion rate > 80%, magnification 100×). (b) Identification of ASCs by detecting cell surface antigens. (c) Representative SEM images of POC-PLA-ASCs patches on the fifth day of cell culture.

The morphology of ASCs cells was observed under inverted microscope, and the stem cell like morphology was observed after the primary ASCs (as shown in Fig 3a). Cell phenotype was identified by flow cytometry. The results showed that the positive expression rates of CD29, CD73, CD90 and CD105 were all higher than 90%, while CD31, CD34, CD44 and CD45 showed negative results (Fig 3b), which indicated the higher purity of ASCs we isolated.

After the preparation of POC-PLA-ASCs scaffolds, abundant ASCs attached on the patches of cell sheets were observed under SEM, as shown in Fig 3c (other SEM pictures and other raw data were shown in S2 File).

### In vivo implantation of the scaffolds in rabbits

The rabbits in each group took few food at the 2^nd^ day, and the food-intake tend to be normal 1 week post operation. The weight of rabbits in each group grows stably. The rabbits received routine antibiotic treatments after chest wall incision surgery and no phenomenon such as swelling, effusion, suppuration and ulceration were observed.

#### Pulmonary function analysis

**Fig 4:**
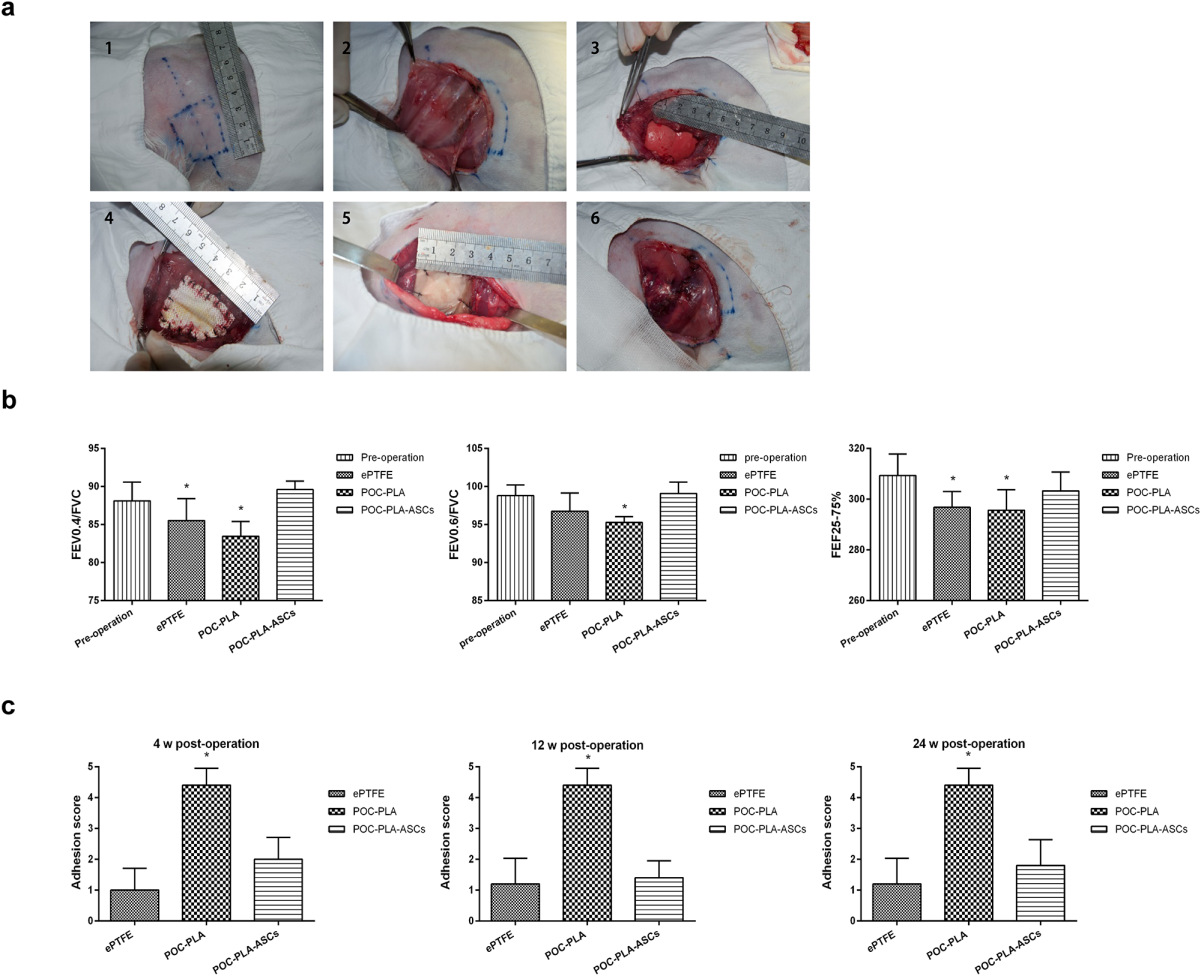
*In vivo* implantation of the scaffolds in rabbits. (a) The representative pictures showed the operating procedure of *in vivo* operations in rabbits. (b) The indexes indicated the pulmonary functions of rabbits from each group at pre-operation and 24 week post-operation. (c) The pleural adhesion score of rabbits from each groups at 4, 12, and 24 week post-operation. *, P<0.05.

At 4, 12, and 24 weeks after surgery (the surgery procedures were as shown in Fig 4a), the results of animal pulmonary function analysis system (AniREs 2005) indicated that there are significant differences between the pulmonary function of rabbit in POC-PLA group and other groups. The difference between sham operation group with ePTFE and POC-PLA-ASCs groups were smaller at middle-late stages post operation (data not shown). While at the 24^th^ week after operation, the rabbits in POC-PLA-ASCs group exhibited better pulmonary functions, which were comparable to the pulmonary functions of rabbits pre-operation (as shown in Fig 4b).

#### Pleural adhesion detection

No obvious ulcer was found in all group untill 24 weeks post operation. Slight adhesive bands between the lung tissue and ePTFE patches which could be separate easily were observed in ePTFE goup. While obvious adhesion were occurred in POC-PLA groups, and the adhesion were dramatically improved in POC-PLA-ASCs groups. The adhesion index score of each group were significantly different (P<0.05) (As shown in Fig 4c).

#### H&E staining

**Fig 5:**
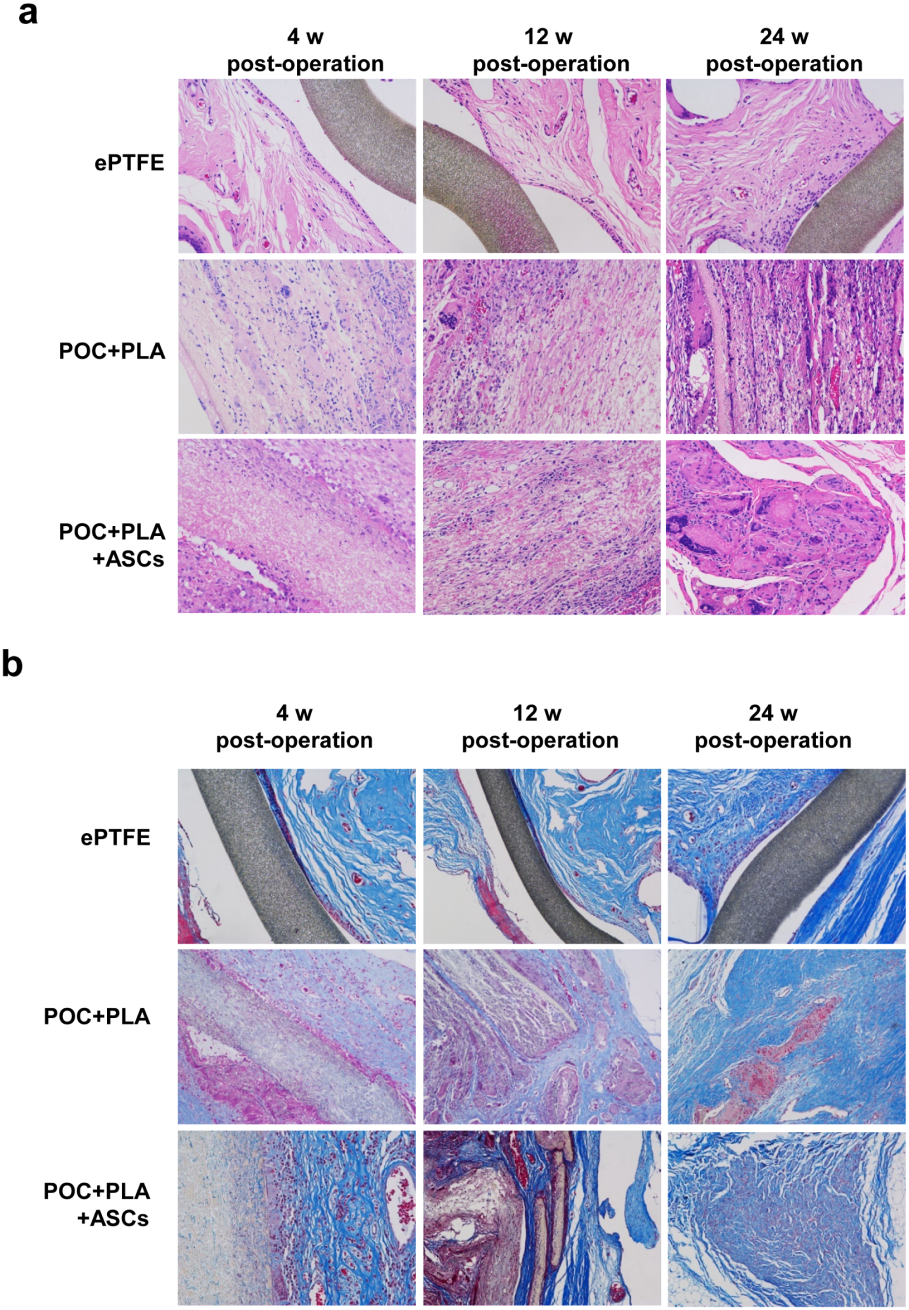
Histological staining of tissues from CWR models. (a) Representative H&E staining images in each group. (b) Representative Masson staining results from each group at 4, 12, and 24 week post-operation (magnification 100×).

As shown in Fig 5a, the ePTFE patches showed no signs of decomposition in 24 weeks, and the direct connection between patches and tissues was loose and easy to be separated. H&E staining showed a bed of dark stained cells, which seemed to be fibroblasts. However, in POC-PLA group, amount of infiltrated cells were observed in patches, and the boundary between tissue and implants was unapparent at 4^th^ week after surgery. At 12 weeks after surgery, more cells were infiltrated into the patches and the patches showed discontinuity resulted from the light stained sheet. At postoperative 24 weeks, a few undegraded POC-PLA filled with cells could still be seen. At postoperative 4 weeks in POC-PLA-ASCs group, amount of cells in materials and plenty of light stained fiber membrane like tissue in the pleural side had been seen. While at 24^th^ week after surgery, the island like fragments among the plenty cell sheet was observed.

#### Masson staining

By application of Masson staining, ePTFE material was sparsely surrounded by mast fibrous tissue. In POC-PLA group, a thick layer of collagenous fiber can be seen at postoperative 4 weeks, while chest muscle interface was gradually covered by fiber at 12^th^ week and the patches were replaced by mast fibers after 24 weeks. While in POC-PLA-ASCs group, several layers of fibers were observed after 4 weeks and the original materials were almost completely replaced by fibrous tissue (Fig 5b).

#### EVG staining

**Fig 6:**
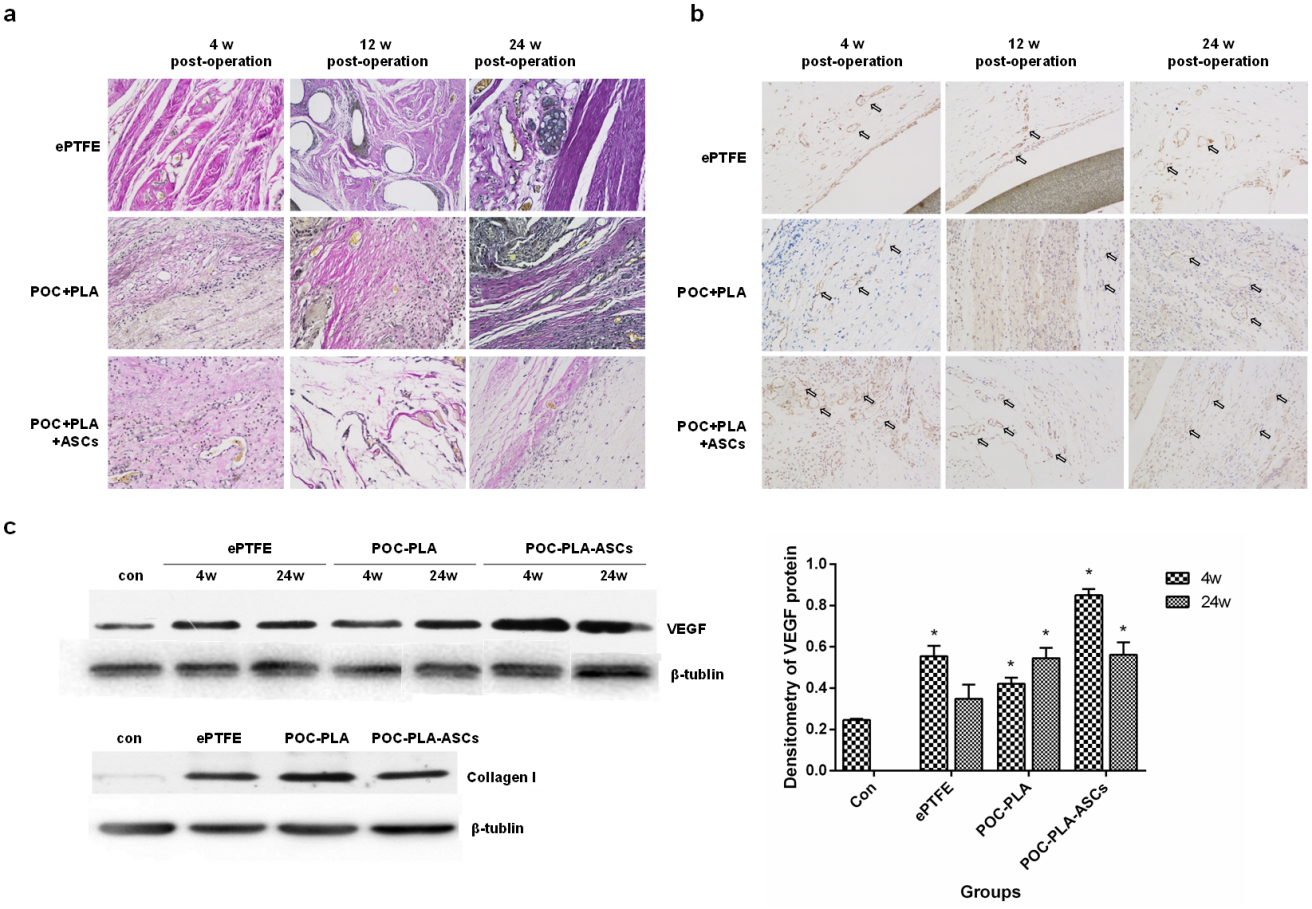
IHC and Western blot for molecular mechanism study. (a) Representative EVG staining results from each group at 4, 12, and 24 week post-operation (magnification 100×). (b) IHC results showed the expression of CD31 in 3 groups. (c) Representative bands of Western blot showed the expression of VEGF in groups at 4 and 24 week post-operation and expression of Collagen I in 3 groups at 24 weeks after operation. The column diagram showed the densitometry of the VEGF Western blot results. *, P<0.05 when compared with the control group.

The EVG staining assay revealed that there was obvious elastic fibers from the 12^th^ week in ASCs group, which was absence in ePTFE and POC-PLA groups (Fig 6a).

#### IHC

As shown in Fig 6b, at postoperative 4 weeks, the blood vessel density around patches in POC-PLA-ASCs group was obvious higher than other groups, among which, ePTFE assumed the lowest vessel density. The vessel density in each group were increased over time, while POC-PLA-ASCs always showed highest vessel density than other groups at different time.

#### Western blot

As shown in Fig 6c, the results of Western blot showed the expression levels of VEGF and Collagen I. Intensity analysis revealed the expression of VEGF in ASCs group at both 4th and 24^th^ week were higher than other groups, and at the 4^th^ week, the expression of VEGF in ASCs group was the highest. The densitometry of the Western blot results were analyzed and shown in Fig 6C, from which we can summarized that the VEGF was up-regulated in ePTFE, POC-PLA and POC-PLA-ASCs groups when compared with control group. While the expression of Collagen I in POC-PLA group were higher than other groups.

## Discussion

Few studies were focus on clinic application of POC. Chung EJ *et al*. fabricated a biodegradable and synthetic tri-component graft consisting of POC, hydroxyapatite nanocomposites (HA) and poly (L-lactide) (PLL), and revealed its good performance in a rabbit ligament reconstruction animal model [23]. The PLA is the most common degradable materials for clinical application. In present study, we constructed a POC-PLA artificial biofilm obtain good elasticity by electrospinning. The biofilm also obtain good three-dimensional structure, as the pore diameter at microscale were observed by SEM. The above characteristics of POC-PLA film are beneficial for ASCs adhesion: ASCs were adhered to the surface of the film 24 h after implantation, while the implants were surrounded by tissues and cells throughout the pores, as well as a large number of new small blood vessels around the film were observed *in vivo* study. All of these finds indicated the patch, as a scaffold for the reconstruction of tissue is feasibility.

In our *in vivo* study, the patches were surrounded by the tissues at the 1^st^ month, which were gradually decomposed at 2^nd^ month and were completely absorbed and replaced by the tissues. It's easy to develop an abnormal thoracic wall during the chest-wall reconstruction in growth-stage. There is no thoracic abnormalities was observed both in ePTFE and POC-PLA group in early stage, while slight sternum shifting was observed in ePTFE reconstruction group at 24^th^ week post-operation. In POC-PLA group, inflammatory cellular infiltration and fibroblast were observed in early stage, and chondrocyte and myofiber like tissues at the edges of material were generated later, which might be the reason for no sternum shifting occurred.

Detection of pulmonary function [24] revealed some differences even though no complications such as respiratory failure was occurred among all of the rabbits. As a clinical common patch, ePTFE have good effects on recovery of postoperative lung function, as the indicators in this group were normal. The sheer POC-PLA patch group showed good reconstructive effect on chest wall, while severe adhesion was occurred at the repair area, which might result to attenuation of the pulmonary ventilation function. Among all of these groups, POC-PLA-ASCs group exhibited excellent effect on recovery of postoperative lung function, the rabbits recovered the normal level of lung function at 24^th^ weeks post operation.

Chest wall adhesion, as one of the normal complications of thoracotomy, could delay the time of operation and increase the incidence of complications for recurrence tumor patients who need reoperation [10]. ePTFE patches showed good adhesion prevention effect, while POC-PLA group was easy to generate adhesion, as various degrees of adhesion were observed in all of the samples in this group. The results of IHC showed high level of TGFβ, IL-6, and TNF-α, indicating the adhesion might be related to the early inflammatory response. However, when the POC-PLA patches were combined with the ASCs, the adhesion was obvious improved. Additionally, POC-PLA patches possessed good cell adhesion ability, which might be the reason of pleura adhesion [25]. On the one hand, the stem cell sheet prevented the direct connection of pleura and material in the early stage, decreased the adsorption abilities of the implants; on the other hand, the cytokines secreted by stem cells might reduce the formation of adhesion in later stage by accelerate the wrapping of material by endothelial cells and fibrocystic.

In conclusion, our present study demonstrated that POC-PLA material was applied for CWR successfully, and ASCs were seeded on the sheets to improve the pleural adhesions and promote the reparation of chest wall defects. It is reasonable to speculate that, as an ideal elastic scaffolds materials, POC-PLA-ASCs will be applicable to a wide range of biological fields.

## Supporting information

S1 File NC3Rs ARRIVE guidelines checklist. (PDF)

S2 File The part of SEM pictures and other raw data. (RAR)
